# The time is now (again) for mpox containment and elimination in Democratic Republic of the Congo

**DOI:** 10.1371/journal.pgph.0003171

**Published:** 2024-06-07

**Authors:** Placide Mbala-Kingebeni, Anne W. Rimoin, Cris Kacita, Laurens Liesenborghs, Jean B. Nachega, Jason Kindrachuk

**Affiliations:** 1 Pathogen Genomics Laboratory, Epidemiology and Global Health Department, Institut National de Recherche Biomédicale, Kinshasa, Democratic Republic of the Congo; 2 Service de Microbiologie, Cliniques Universitaires, Faculté de Médecine, Université de Kinshasa, Kinshasa, Democratic Republic of the Congo; 3 Department of Epidemiology, UCLA Fielding School of Public Health, Los Angeles, California, United States of America; 4 UCLA-DRC Health Research and Training Program, UCLA Fielding School of Public Health, Los Angeles, California, United States of America; 5 Monkeypox and Viral Hemorrhagic Fever Control Program, Ministry of Public Health, Kinshasa, Democratic Republic of the Congo; 6 Department of Clinical Sciences, Institute of Tropical Medicine, Antwerp, Belgium; 7 Department of Microbiology, Immunology and Transplantation, KU Leuven, Leuven, Belgium; 8 Departments of Epidemiology, Infectious Diseases and Microbiology and Center for Global Health, University of Pittsburgh School of Public Health, Pittsburgh, Pennsylvania, United States of America; 9 Departments of Epidemiology and International Health, Johns Hopkins Bloomberg School of Public Health, Center for Global Health, Johns Hopkins University Baltimore, Baltimore, Maryland, United States of America; 10 Department of Medicine, Division of Infectious Diseases, Stellenbosch University Faculty of Medicine and Health Sciences, Cape Town, South Africa; 11 Department of Medical Microbiology & Infectious Diseases, Max Rady College of Medicine, University of Manitoba, Winnipeg, Canada; 12 Department of Internal Medicine, Max Rady College of Medicine, University of Manitoba, Winnipeg, Canada; Emory University School of Medicine, UNITED STATES

## Background

Mpox, a zoonotic disease caused by monkeypox virus (MPXV), was first identified in humans in the Democratic Republic of the Congo (DRC) in 1970 [1–3]. MPXV is subclassified into clade I—formerly Congo Basin (Central Africa) clade, and clade II—formerly West African clade. Clade II comprises two subclades: IIa and IIb, the latter being responsible for the recent global epidemic [4, 5]. Several studies have suggested that clade I infections are associated with greater disease severity compared to clade II [6, 7].

## Global expansion of mpox

Rapid geographic expansion of MPXV across non-endemic regions of the globe resulted in the first global mpox epidemic from 2022–2023 and the declaration of a public health emergency of international concern by the World Health Organization (WHO). While zoonosis has historically been the primary driver of infections in humans with limited secondary infections through human-to-human contact, >90% of infections were linked to secondary transmission during the 2022 global epidemic, mainly through sexual contact among gay, bisexual, or other men who have sex with men (GBMSM). These observations could represent: i) a recent transition in MPXV transmission and clinical presentation from that seen historically; ii) greater diversity in clinical presentation and transmission than previously described; or iii) a combination thereof.

## Redefining mpox in DRC

In recent years there has been a sharp increase in mpox cases in DRC, nearly doubling from 2021 to 2022 and more than doubling from 2022 to 2023 with nearly 15,000 suspected mpox cases and more than 600 deaths in 2023 (case fatality rate of 4.5%) [8]. Concerningly, early epidemiological data from 2024 suggested an increasing disease burden as compared to the same period in 2023. This has been accompanied by continued geographic expansion of the virus in DRC, including to large urban centers (e.g. Kinshasa, pop. 17 million). While most mpox emergence events have been due to zoonoses with rodent species being the presumed reservoir, recent outbreaks have demonstrated increasing trends of human-to-human transmission. Further, suspected cases during the ongoing DRC mpox outbreak have been found among sex workers and GBMSM [9]. While the greatest burden for mpox morbidity and mortality continues to be found among children <15, data from suspected cases in 2023 suggest that there are increasing case burdens among women compared to men, with ~1/3 of recent cases in South Kivu being associated with female sex workers aged 20–29 years [10, 11].

We recently reported the first confirmed cluster of sexual transmission-associated clade I MPXV infections [9]. Our investigation demonstrated that sexual transmission is conserved across clade I and II MPXV and highlighting the broader risks for geographic expansion of the virus to new global regions such as seen during clade IIb mpox epidemic in 2022. However, there is no current data regarding clade I-associated mpox infections morbidity and mortality including among key populations that were found to be at increased risk for mpox in 2022 including sex workers and those that identify as GBMSM. Given the high sequence homology across clade I and II MPXV, the increasing frequencies of human-to-human transmission seen during recent outbreaks, and the risks for misdiagnosis and treatment due to atypical clinical presentation, there is a critical need to characterize clade I mpox morbidity and mortality related to sexual transmission, with a particular focus on those at increased risk for infection.

## Existing knowledge gaps

**Fig 1:**
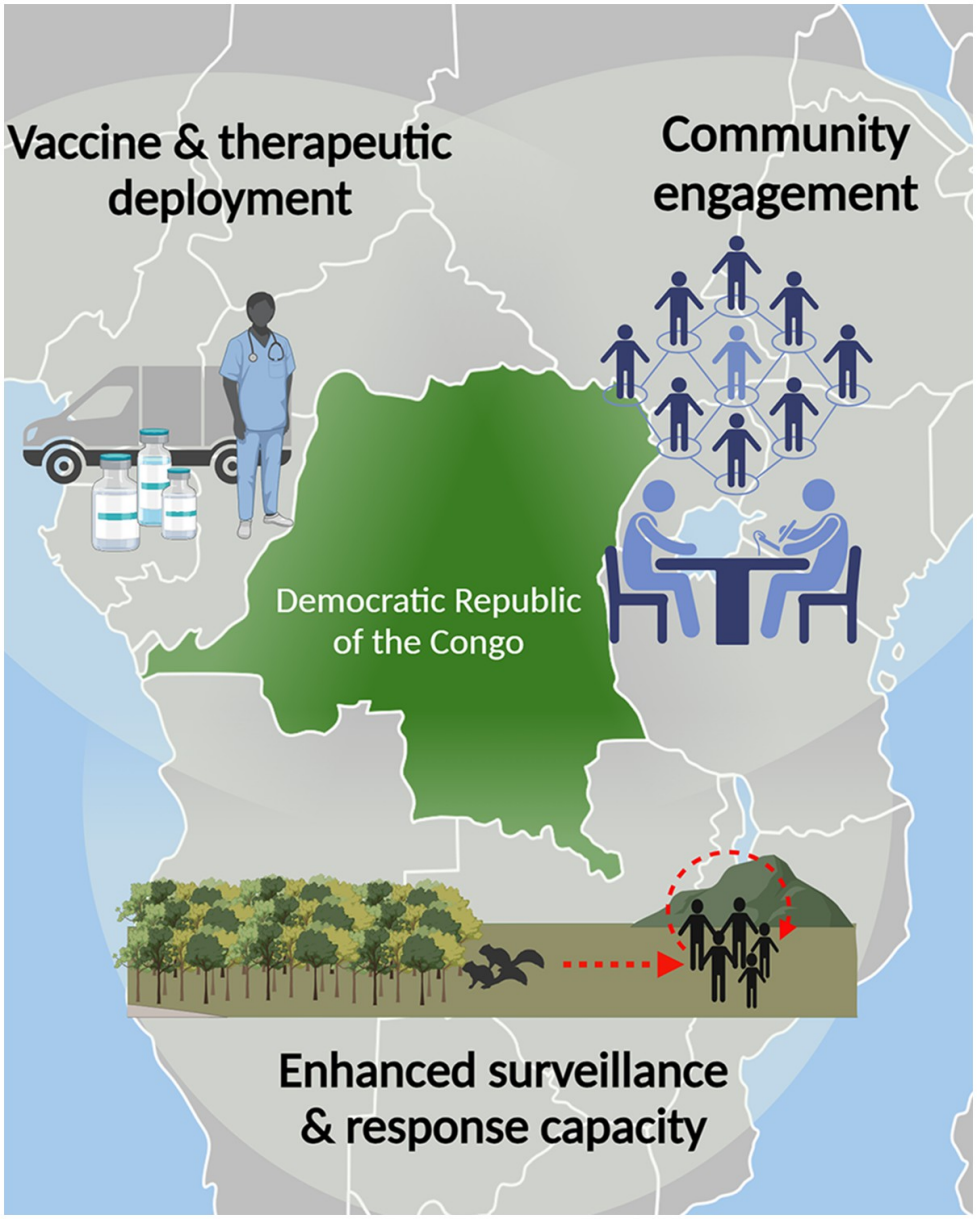
Key focal areas for mpox containment and mitigation in the DRC. Image produced with BioRender. Map image provided by Servier Medical Arts licensed under CC BY 4.0.

There is an urgent need to define mpox research priorities given the ongoing outbreak in DRC. Of paramount importance is sustained global investment in surveillance and response capacity. Given the increasing complexity surrounding mpox transmission, key research and response considerations include (Fig 1):

- Increased prioritization of collaborative research and clinical partnerships across endemic regions
- Enhanced surveillance and response capacities in vulnerable regions
- Equitable access to vaccines and therapeutics in endemic and vulnerable regions
- Increased integration of social sciences into outbreak preparedness and response efforts
- Clinical characterization of mpox across exposure settings and transmission routes

## Mpox containment and mitigation in DRC

While vaccination campaigns were expedited across numerous non-endemic regions during the global epidemic, there are no active vaccination campaigns within mpox endemic regions of Africa. Prior work from Petersen and colleagues highlighted the existing hurdles for mpox vaccine clinical trials and deployment in DRC [12]. The recent licensure and deployment of third-generation vaccines during the global mpox epidemic have provided extensive real-world data regarding vaccine safety and efficacy. Nigeria was the first to license MVA-BN after working closely with both the European Medicines Agency (EMA and the US Food and Drug Administration (FDA). The DRC Ministry of Health, the drug authorities (Autorité Congolaise de Réglementation Pharmaceutique; ACOREP) and National Immunization Technical Advisory Groups (NITAG) similarly expressed interest in licensing both MVA-BN and LC16. The EMA and FDA review and licensure could be used to facilitate review in the DRC. From a logistical perspective, rapid vaccine deployment strategies must consider not only the acquisition of vaccine doses but also transportation to key mpox circulation hotspots, cold-chain storage, and community engagement/knowledge mobilization strategies to ensure uptake among key populations at highest risk for severe disease or infection. While discussions are ongoing between the DRC, vaccine suppliers, and international organizations, a firm agreement on vaccine deployment has yet to be enacted. Given the increasing burden of mpox in endemic regions, there is a critical need for both regional and international partners to support vaccination campaigns and ensure vaccine accessibility and equity considerations. We recently modeled the potential impacts of MVA-BN vaccination in DRC to estimate the impact of vaccination strategies on mpox burden with consideration for historic burdens of the disease across all provinces and age groups within DRC [13]. Modeling suggested that vaccination of 80% of children <15 years in DRC would result in the greatest reductions on mpox circulation. However, given the increasing identification of sexual transmission-related cases within DRC, this and other vaccination modeling strategies will need to factor in the burden of sexual transmission on mpox cases in DRC to address the impacts of targeted vaccination and therapeutic campaigns on key populations. It should be appreciated that the DRC has leading expertise in emerging infectious disease response activities including resurgent Ebola virus disease outbreak which have resulted in rapid vaccine deployment and vaccination campaigns as well as extensive contact tracing, including during the COVID-19 pandemic [14, 15]. This has included broad knowledge mobilization campaigns.

There is a paucity of data regarding the role of sexual transmission in the ongoing mpox outbreak in DRC, morbidity and mortality estimates for sexually acquired clade I mpox, and relative infection risks within key populations. Further, longitudinal serosurveillance studies are critically needed to more accurately determine the exposure burdens across communities, including those where stigma or discrimination could preclude self-reporting or health-seeking behavior. Recent suspected mpox among sex workers in Kinshasa (3/21 confirmed cases in Kinshasa) raises concerns regarding increasing risks for much broader human-to-human transmission within large urban centers. Recent mpox cases in Republic of Congo, with regular transit between the capital Brazzaville and Kinshasa, highlight the need for increased surveillance and data sharing partnerships. While the global epidemic garnered international spotlight and renewed interest in mpox, threat reduction must include concerted efforts for control efforts in endemic regions.

## References

[1] Breman JG, Kalisa R, Steniowski MV, Zanotto E, Gromyko AI, Arita I. Human monkeypox, 1970–79. Bull World Health Organ. 1980;58(2):165–82. Epub 1980/01/01. . 6249508 PMC2395797

[2] Foster SO, Brink EW, Hutchins DL, Pifer JM, Lourie B, Moser CR, et al. Human monkeypox. Bull World Health Organ. 1972;46(5):569–76. Epub 1972/01/01. . 4340216 PMC2480784

[3] Ladnyj ID, Ziegler P, Kima E. A human infection caused by monkeypox virus in Basankusu Territory, Democratic Republic of the Congo. Bull World Health Organ. 1972;46(5):593–7. Epub 1972/01/01. . 4340218 PMC2480792

[4] Ulaeto D, Agafonov A, Burchfield J, Carter L, Happi C, Jakob R, et al. New nomenclature for mpox (monkeypox) and monkeypox virus clades. Lancet Infect Dis. 2023;23(3):273–5. Epub 2023/02/10. doi: 10.1016/S1473-3099(23)00055-5 . 36758567 PMC9901940

[5] Thornhill JP, Barkati S, Walmsley S, Rockstroh J, Antinori A, Harrison LB, et al. Monkeypox Virus Infection in Humans across 16 Countries—April-June 2022. N Engl J Med. 2022;387(8):679–91. Epub 2022/07/23. doi: 10.1056/NEJMoa2207323 . 35866746

[6] Weaver JR, Isaacs SN. Monkeypox virus and insights into its immunomodulatory proteins. Immunol Rev. 2008;225:96–113. Epub 2008/10/08. doi: 10.1111/j.1600-065X.2008.00691.x . 18837778 PMC2567051

[7] McCollum AM, Damon IK. Human monkeypox. Clin Infect Dis. 2014;58(2):260–7. Epub 2013/10/26. doi: 10.1093/cid/cit703 . 24158414 PMC5895105

[8] MSPHP. REPORT ON THE EPIDEMIOLOGICAL SITUATION OF MONKEYPOX VIRUS—S1-S6 2024. Ministry of Public Health, Hygiene and Prevention of the Democratic Republic of the Congo, 2024.

[9] Kibungu EM, Vakaniaki EH, Kinganda-Lusamaki E, Kalonji-Mukendi T, Pukuta E, Hoff NA, et al. Clade I-Associated Mpox Cases Associated with Sexual Contact, the Democratic Republic of the Congo. Emerg Infect Dis. 2024;30(1):172–6. Epub 2023/11/29. doi: 10.3201/eid3001.231164 . 38019211 PMC10756366

[10] World Health Organization. Disease Outbreak News; Mpox (monkeypox) in the Democratic Republic of the Congo. 23 November 2023. https://www.who.int/emergencies/disease-outbreak-news/item/2023-DON493.

[11] Vakaniaki EH, Kacita C, Kinganda-Lusamaki E, O’Toole A, Wawina-Bokalanga T, Mukai-Bamuleka D, et al. Sustained Human Outbreak of a New MPXV Clade I Lineage in Eastern Democratic Republic of the Congo. medRxiv. 2024. Epub 14 April 2024. doi: 10.1101/2024.04.12.24305195 PMC11485229 38871006

[12] Petersen BW, Kabamba J, McCollum AM, Lushima RS, Wemakoy EO, Muyembe Tamfum JJ, et al. Vaccinating against monkeypox in the Democratic Republic of the Congo. Antiviral Res. 2019;162:171–7. Epub 2018/11/18. doi: 10.1016/j.antiviral.2018.11.004 . 30445121 PMC6438175

[13] Savinkina A K K, Bogoch II, Rimoin AW, Hoff NA, Shaw SY, Mbala-Kingebeni P, Gonsalves GS. Modeling Vaccination Approaches for Mpox Containment and Mitigation in the Democratic Republic of the Congo. SSRN. 2024. Epub 18 March 2024. 10.2139/ssrn.4759169. 39393385

[14] United Nations. Ebola vaccination campaign begins in DR Congo to counter new outbreak. UN News Global perspective Human stories: 27 Apr 2022. Ebola vaccination campaign begins in DR Congo to counter new outbreak | UN News.

[15] Adepoju P. Ebola and COVID-19 in DR Congo and Guinea. Lancet Infect Dis. 2021;21(4):461. Epub 2021/03/28. doi: 10.1016/S1473-3099(21)00155-9 . 33773127 PMC7990479

